# Beyond regulations at DNA levels: A review of epigenetic therapeutics targeting cancer stem cells

**DOI:** 10.1111/cpr.12963

**Published:** 2020-12-13

**Authors:** Shunhao Zhang, Yanji Gong, Chunjie Li, Wenbin Yang, Longjiang Li

**Affiliations:** ^1^ State Key Laboratory of Oral Disease National Clinical Research Center for Oral Disease Department of Oral and Maxillofacial Surgery West China Hospital of Stomatology Sichuan University Sichuan Province Chengdu China; ^2^ State Key Laboratory of Oral Disease National Clinical Research Center for Oral Disease Department of Temporomandibular Joint West China Hospital of Stomatology Sichuan University Chengdu Sichuan Province China; ^3^ State Key Laboratory of Oral Disease National Clinical Research Center for Oral Disease Department of Head and Neck Oncology West China Hospital of Stomatology Sichuan University Chengdu Sichuan Province China

**Keywords:** cancer stem cells, DNA methyltransferase, epigenetic therapeutics, histone deacetylase, miRNA

## Abstract

In the past few years, the paramount role of cancer stem cells (CSCs), in terms of cancer initiation, proliferation, metastasis, invasion and chemoresistance, has been revealed by accumulating studies. However, this level of cellular plasticity cannot be entirely explained by genetic mutations. Research on epigenetic modifications as a complementary explanation for the properties of CSCs has been increasing over the past several years. Notably, therapeutic strategies are currently being developed in an effort to reverse aberrant epigenetic alterations using specific chemical inhibitors. In this review, we summarize the current understanding of CSCs and their role in cancer progression, and provide an overview of epigenetic alterations seen in CSCs. Importantly, we focus on primary cancer therapies that target the epigenetic modification of CSCs by the use of specific chemical inhibitors, such as histone deacetylase (HDAC) inhibitors, DNA methyltransferase (DNMT) inhibitors and microRNA‐based (miRNA‐based) therapeutics.

## Graphical abstract

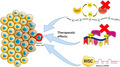

## 1 INTRODUCTION

Cancer is one of the leading fatal diseases that severely threaten human life.^1^, ^2^ Approximately 18 million people are diagnosed with cancer every year and 9.6 million will ultimately die of cancer.^3^ However, traditional therapeutics are effective only for few malignant tumours^4^ due to metastasis, recurrence, heterogeneity, resistance to chemotherapy and radiotherapy, and escape from immunological surveillance,^5^ all of which might be explained by the properties of cancer stem cells (CSCs).^6^ Initial studies indicating that cancer cells may have similar stem‐like characteristics were conducted in teratomas,^7^ which led to the first CSC hypothesis that tumours consist of malignant stem cells and their benign progeny,^8^ and eventually the identification of a small population of leukaemia stem cells initiating leukaemia in mice.^9^ CSCs, currently defined as initiating tumour cells, have been identified in various cancer types and are regarded as one of the most promising targets for cancer therapeutics because of their intrinsic potential to cause cancer initiation, relapse, metastasis, multidrug resistance and radiation resistance.^10^

However, this level of cellular plasticity cannot be entirely explained by irreversible genetic alterations. Thus, the significance of reversible epigenetic modifications has gradually been discerned in terms of the activation of specific transcriptional networks underlying the diverse cellular states of CSCs. Epigenetic changes are covalent modifications to DNA or histones without altering the DNA sequence, including DNA methylation, histone modification (methylation, acetylation, phosphorylation), and non‐coding RNA (ncRNA) expression.^11^ The main types of epigenetic modifications that have been targeted by cancer treatment in recent years are DNA methylation, histone acetylation and ncRNA expression. Moreover, the gene expression patterns triggered by exact epigenetic modulations are unique to CSCs. Therefore, selective epigenetic tumour therapeutics based on a deeper understanding of epigenetic alterations will definitely benefit the development of novel cancer treatments.

## 2 CSCs AND THEIR ROLE IN CANCER INITIATION AND PROGRESSION

Human cancer is a type of genetic disease that originates from a series of accumulating mutations or genomic alterations, some of which are only found in specific cancer types, such as c‐KIT mutations in gastrointestinal stromal cancers, whereas mutations in TP53 occur in almost every type of cancer. These aberrant gene expressions eventually affect different pathways regulating cell signalling, cell growth, DNA repair and other cellular events leading to several changes in normal cells such as the acquisition of the ability to divide infinitely, aid angiogenesis, escape from growth‐inhibitory signals, evade apoptosis, and promote invasion and metastasis.^12^, ^13^, ^14^ The classical view of tumorigenesis argues that the majority of tumour cells are capable of proliferating extensively and initiating new cancer cells on their own. However, such points of view are unsatisfactory because they cannot explain the few colonies observed in vitro and the need for a large number of tumour cells to form new tumour cells in vivo.^15^ Given these unexplained properties of cancer cells, studies are continuously being conducted and a large body of work has deepened our knowledge regarding tumorigenesis. A more comprehensive understanding of cancer was obtained after consensus of the fact that tumour cells are heterogeneous, suggesting that only a limited subset of cells have the potential to fuel cancer initiation and progression, which was first proven in acute myeloid leukaemia (AML).^9^, ^16^ Furthermore, subsequent research identified a small number of malignant stem cells with the ability to initiate solid tumours in mammary cancers.^17^

These malignant cells are termed CSCs in accordance with their stemness or stem‐like properties, including the capabilities of differentiation and self‐renewal extensively shared with normal stem cells. In addition, the decisive difference between CSCs and normal stem cells is their potent tumour‐initiation capacity, indicating the significance of eliminating all CSCs in order to achieve effective treatment.^17^ Apart from the shared stem‐like properties, another common characteristic is the similar signalling pathways collectively utilized by these two kinds of stem cells, which highlights the importance of specific signalling pathways in the course of cancer initiation and progression.^18^, ^19^

### 2.1 Cancer initiation

CSCs regularly serve as a small population of primary cells that fuel the initiation of diverse types of solid cancers.^20^ Taking the initiation of head and neck squamous cell carcinoma (SCC) as an example, this course can only be triggered by gene mutations, such as those that cause the overexpression of Kras and p53 or affect the Tgfb and Pten signalling pathways, in undifferentiated stem‐like cells of the epithelium.^21^, ^22^ Likewise, the aberrant expression of oncogenes, such as Sox2 and Stat3, in undifferentiated basal cells leads to oesophageal SCC initiation; however, this does not affect differentiated cells.^23^ In colon cancer, the downregulation of APC associated with Wnt signalling and subsequent activation of Ras and phosphoinositide 3‐kinase signalling results in cancer development. The low rate of these mutations and the time needed for the process of cancer development are almost certainly due to the hallmarks of CSCs.^24^, ^25^ Although all of these studies collectively suggest that mutations in CSCs may lead to cancer initiation, it is noteworthy that differences in the cells that were originally mutated are likely to have a paramount impact on cancer type. For example, in breast tumour models established in mice, loss of p53 along with BRCA1 in basal stem cells resulted in the development of malignant adenomyoepitheliomas, which is a tumour type rarely seen in human breast cancer patients, whereas the same mutations in luminal progenitors led to adenocarcinomas.^26^ In conclusion, both mutated genes and the original cells are decisive for tumour type.

### 2.2 Epithelial to mesenchymal transition, cancer metastasis and chemotherapy drug resistance

CSC and stem cell signals play important roles in cancer metastasis,^27^ according to which CSCs undergo the process of epithelial to mesenchymal transition (EMT) and obtain the capability to transfer from the primary site to distal tissues or organs. In general, EMT is a continuous process that reduces adhesion between cells at first and then decreases cell polarity and enhances cell motility, and finally provides CSCs with invasive mesenchymal properties.^28^ The EMT process of CSCs is generally associated with intrinsic epigenetic changes in these cancer‐initiating cells. For example, the chromatin of genes fuelling EMT is more reachable and active in SCC stem cells derived from hair follicle stem cells in comparison with the less metastatic, more differentiated cell populations of SCC arising from epidermal stem cells.^29^ Moreover, studies have revealed that CSCs with EMT features are more resistant to chemotherapy drugs than other cancer cells.^30^ Traditional chemotherapy drugs such as cisplatin, gemcitabine and 5‐fluorouracil are less effective in pancreatic tumour cell lines with an EMT‐like phenotype.^31^ The increased chemotherapeutic drug resistance is mainly mediated by the overexpression of drug efflux transporters such as multidrug resistance protein 1 (MDR1), multidrug resistance‐associated protein 1 (MRP1), and ATP‐binding cassette sub‐family G member 2 (ABCG2), whose function is to expel drugs from cells using ATP against concentration gradients.^32^, ^33^ The overexpression of these transporters is likely caused by various pathways and mechanisms encompassing epigenetic changes, which are attracting increasing attention. For instance, the downregulation of histone deacetylase 1 (HDAC1) or increased H3S10 phosphorylation, H3K4 tri‐methylation and histone H3 acetylation can all lead to the activation of ABCG2 transcription, finally resulting in enhanced drug efflux capability,^34^ suggesting that epigenetic alterations may be potential targets for cancer treatments.

## 3 EPIGENETIC REGULATION OF CSCS

There is no doubt that DNA encodes all the biological information essential to living creatures, whose mutations may lead to alterations in cellular differentiation and improper development. DNA inside the nucleus, packaged into chromatin, forms a compact nucleoprotein structure in which the nucleosome is the smallest functional unit, composed of 147 base pairs of DNA wrapped around a core of eight histone proteins.^35^ This octamer consists of two copies each of the H2A, H2B, H3 and H4 proteins whose amino‐terminal lysine‐rich tails protrude out of the nucleosome and potentially provide targets for post‐translational modifications. In addition, approximately 50 base pairs of linker DNA packaged by the linker histone protein H1 separates adjacent nucleosomes.^36^ Thus, apart from regulations at the DNA level, there are diverse epigenetic modulations of gene expression that play a vital role in the initiation, proliferation, metastasis and invasion of CSCs. Epigenetics is traditionally defined as reversible and hereditable changes in the level of gene expression without affecting DNA sequence or associated factors, and the processes of which have an impact on different stages of gene expression such as transcription, post‐transcription, translation and post‐translation.^37^ Here, we discuss normal epigenetic regulation in terms of three interrelated processes: chromatin modification mainly referring to histone acetylation, DNA methylation and regulation of ncRNA expression.

### 3.1 Histone acetylation

Playing an active role in the regulation of cellular processes such as cell differentiation, proliferation, angiogenesis and apoptosis, acetylation is the most common modification among these epigenetic changes. As a consequence, aberrant acetylation is believed to be relevant to various cellular events in cancer pathologies; notably, the global hypoacetylation of H4 is one of the most common hallmarks of cancer.^38^

**FIGURE 1:**
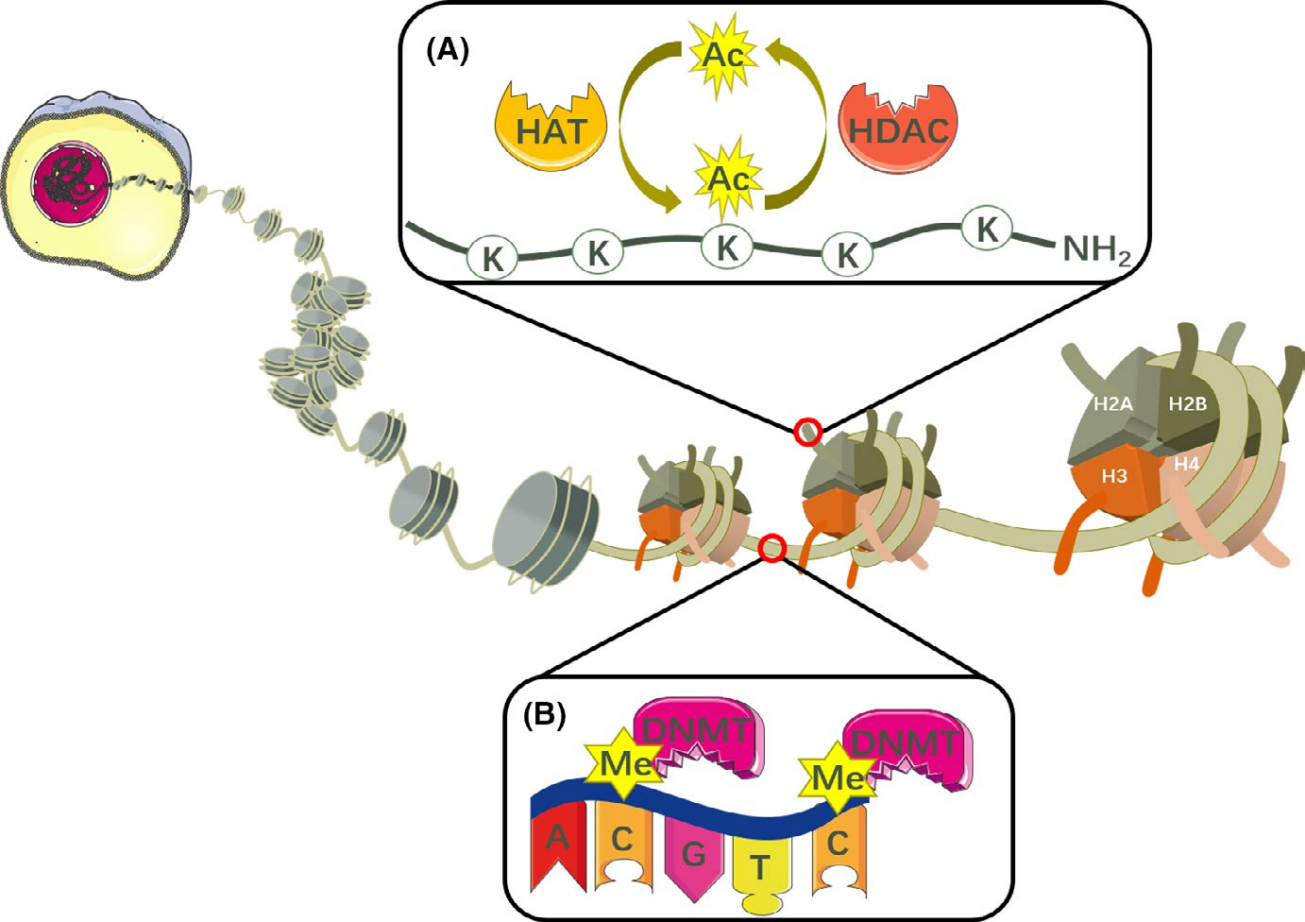
(A) Nucleosomes encompass eight histone proteins including two each of H2A, H2B, H3 and H4. The lysine residues in the amino‐terminal tails of histones protruding from the octamer can either be acetylated by HATs or be deacetylated by HDACs. (B) DNA can also be epigenetically modified by DNMT‐based methylation. This process is mediated by several DNMTs such as DNMT1, DNMT3A and DNMT3B through catalysing a methyl group to CpG dinucleotides. K: lysine residues; AC: acetyl group; Me: methyl group

The level of histone acetylation is mainly regulated by HDACs and histone acetyltransferases (HATs). HATs can acetylate lysine residues in histone tails, while HDACs can remove an acetyl group from the ε‐amino groups of lysine residues (Figure 1A). Therefore, the positive charge in the N‐terminal region of histone cores increases, interactions with negatively charged DNA are consolidated, and the access of transcriptional machinery to the DNA template is blocked, resulting in gene silencing.^39^ Based on sequence homologies, the HDACs identified to date can be divided into four classes. HDAC1, 2, 3 and 8 are class I. Class II HDACs can be further divided into two classes, including class IIa (HDAC4, 5, 7, and 9) and IIb (HDAC6 and 10). Class III HDACs are also known as sirtuins (sirt1‐7), and HDAC11 is class IV.^40^ Several abnormal cellular events such as dysfunction of the cell cycle, cell growth, chromatin decondensation, cell differentiation, apoptosis and angiogenesis have been observed in several cancer types after silencing or inhibiting HDACs.^41^ Meanwhile, it is widely acknowledged that HDACs are overexpressed in tumour cells.^42^ The upregulation of HDAC1 is observed in breast, colon, oesophageal, lung, gastric and prostate cancers. HDAC2 is upregulated in gastric, cervical and colorectal cancers; HDAC3 is upregulated in breast and colon cancers; and HDAC6 is upregulated in breast cancer.^42^, ^43^, ^44^, ^45^

### 3.2 DNA methylation

As a result of abundant studies on DNA methylation, it is widely acknowledged that cancer origin and progression are closely associated with aberrant DNA methylation,^46^ which is routinely mediated by DNA methyltransferases (DNMTs) through the catalysis of a methyl group from S‐adenosyl methionine (SAM) to the carbon‐5 position of the cytosine ring, and finally producing S‐adenosyl‐l‐homocysteine (SAH)^47^ (Figure 1B). Studies regarding the DNMTs DNMT1, DNMT3A and DNMT3B are currently ongoing. Typically, DNMT1 almost certainly maintains DNA methylation during DNA replication,^48^ whereas DNMT3A and DNMT3B are prone to de novo DNA methylation by catalysing the methylation of unmethylated DNA, with the assistance of the catalytically inactive DNMT3 L.^49^, ^50^ It is noteworthy that hypermethylated CpG islands are observed in or near promoter regions, whereas gene bodies become hypomethylated in tumours with abnormal methylation.^51^ Various types of mutations in DNMTs contribute to divergent routes of aberrant DNA methylation. Taking initiating mutations as an example, more than 20% of samples derived from patients suffering from AML had mutations in DNMT3A. In addition, more than half of the mutations occurred at amino acid R882,^52^ which were later confirmed to be dominant‐negative, leading to decreased catalytic activity of DNMT3A and focal hypomethylation, whereas wild‐type DNMT3A R882 showed a hypermethylation pattern in AML DNA.^53^, ^54^ DNMT3A with initiating mutations is capable of creating an ancestral or founder preleukaemic clone, establishing an environment suitable for additional mutations forming malignant clones.^55^, ^56^ Then, subclones of overt leukaemia arise, providing that oncogenes have undergone further mutation.^57^, ^58^, ^59^ In addition, ancestral clones are consistently present both when patients are in remission or suffering from relapse.^60^

### 3.3 Regulation of ncRNAs

NcRNAs, including microRNAs (miRNAs), long non‐coding RNAs (lncRNAs) and circular RNAs (circRNAs), are a class of transcribed RNA molecules that do not encode proteins. However, they are involved in many biological processes by regulating gene expression and interacting with chromatin, proteins, and other coding and non‐coding RNAs.

#### 3.3.1 MiRNAs

**FIGURE 2:**
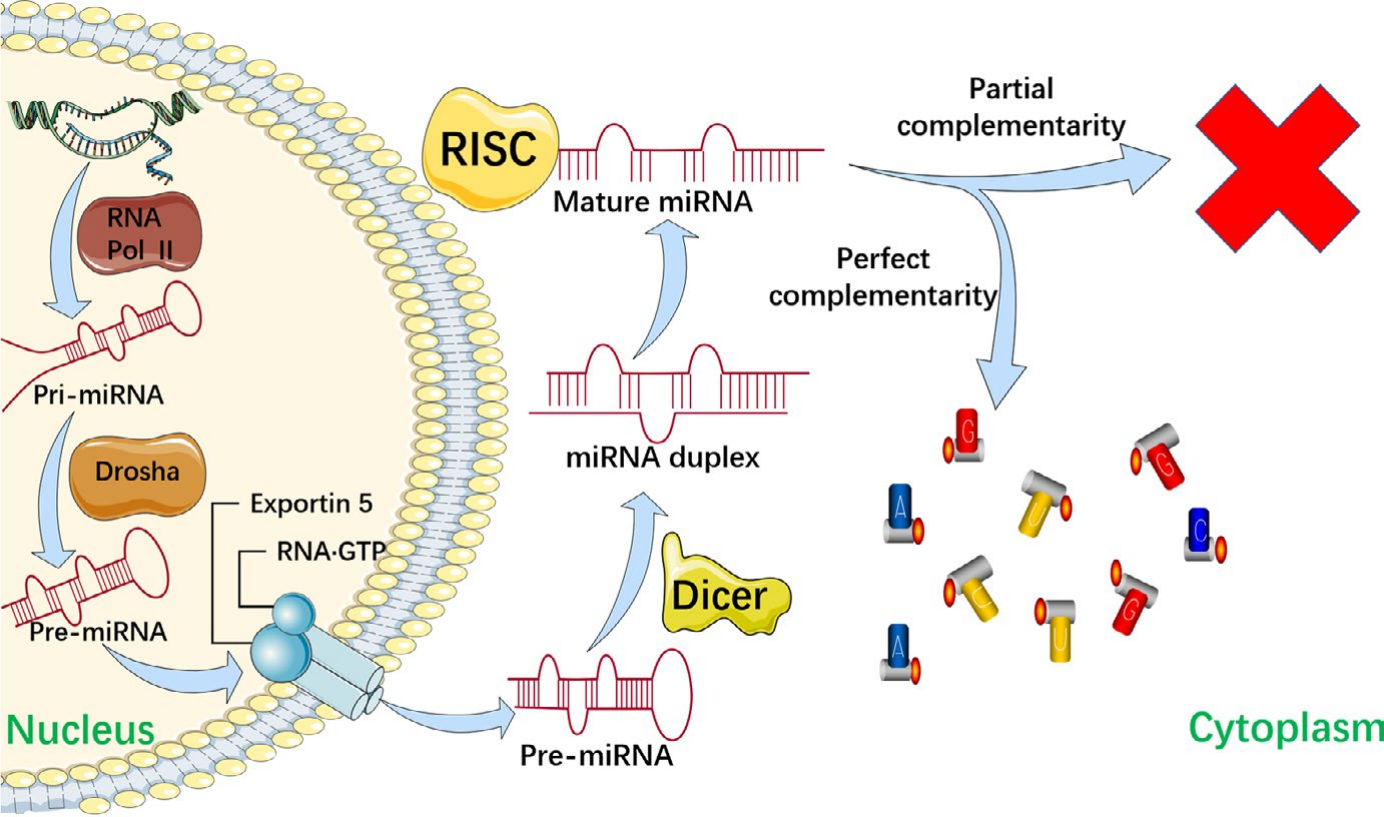
The miRNA biogenesis begins with their transcription by RNA polymerase II which produces primary miRNA (pri‐miRNA) as an end product. Then a type III RNase Drosha along with its cofactor protein DGCR8 binds to the pre‐miRNA to generate precursor miRNA (pre‐miRNA) by mediating enzymatic cleavages. And the pre‐miRNA is exported to the cytoplasm via the exportin 5–RNA•GTP complex. Next, the RNase III Dicer binds to the pre‐miRNA to cut the terminal loop which generates miRNA duplex. In the next step, the RNA‐induced silencing complex (RISC) is incorporated by the miRNA duplex mediated by the AGO family proteins. Depending on whether the mature miRNA is partially or perfectly complementary to the target mRNA, this leads to inhibited translation or degradation, respectively

MiRNAs, which consist of highly conserved, 18‐24 nucleotide sequences,^61^ are a major subtype of ncRNA that are actively involved in central biological processes such as cell differentiation, proliferation and survival by sequence‐specifically blocking mRNA translation, leading to translational degradation or inhibition.^62^ It is widely acknowledged that aberrant expression of miRNAs is closely related to various diseases such as cardiovascular diseases, hepatitis, and cancer. Generally, there are two main explanations for the dysregulation of miRNAs: (1) downregulation of enzymes such as argonaute 2 (AGO2), Dicer, exportin 5, and Drosha, which modulate miRNA biogenesis; and (2) genomic events such as mutations, deletion amplification or transcriptional changes^62^, ^63^, ^64^, ^65^ (Figure 2).

MiRNAs are regarded as imperative components of epigenetic processes, alongside DNA methylation and histone modification. Intriguingly, miRNAs are believed to regulate epigenetic modifiers such as HDACs and DNMTs, and are targeted by epigenetic modifications such as DNA methylation.^66^, ^67^ On the one hand, some miRNAs, known as epi‐miRNAs, can directly or indirectly affect the expression of various epigenetic regulators. For instance, restoring the expression of the tumour suppressor miRNA‐140, which is downregulated in osteosarcoma (OS), prevents tumour cells from proliferation via HDAC4.^68^ In addition, the miRNA‐29 family was shown to play a positive role in the re‐expression of silenced tumour suppressor genes by complementing with the 3’‐untranslated region (3’‐UTR) of DNMT3A and DNMT3B.^67^ On the other hand, the regulation of miRNA expression by DNA methylation, for example, increased levels of miRNA‐370 and decreased levels of β‐catenin downstream targets, which eventually led to inhibition of colony formation and cell proliferation ability, as observed after OS cells were treated with the DNMT inhibitor decitabine.^69^ These results indicate that the expression of tumour suppressor miRNAs may be activated by DNA demethylation.

#### 3.3.2 LncRNAs

LncRNAs are a subtype of ncRNA that lack an open reading frame and are longer than 200 base pairs.^70^ Importantly, they can mediate chromatin and transcriptomic alterations in various cancer phenotypes. For instance, the poor prognosis and recurrence of prostate and breast tumours are associated with aberrant expression of lncRNA metastasis‐associated lung adenocarcinoma transcript 1 (MALAT1).^71^ In addition, the lncRNA BORG (BMP/OP‐responsive gene) can improve metastatic outgrowth of dormant breast‐disseminated cancer cells by inhibiting transcription through the E3 SUMO ligase TRIM28.^72^ In brief, lncRNAs have functional significance in terms of driving cancer progression and recurrence.

#### 3.3.3 CircRNAs

CircRNAs are a single‐stranded closed circular RNAs that lack 5’‐3’ ends and poly (A) tails.^73^ In recent years, there have been an increasing number of reports that circRNAs might be related to the pathogenesis of silicosis,^74^ diabetes,^75^ osteoarthritis,^76^ Alzheimer's disease,^77^ cardiovascular diseases,^78^ nervous system diseases ^79^ and tumours.^80^ In cancer, circRNAs are widely believed to be involved in cancer cell proliferation, metastasis, and stemness ^81^ through the following mechanisms: (1) acting as miRNA sponges and RNA‐binding proteins sponges to regulate gene expression. For example, circRNA ciRS‐7 can serve as a strong miRNA sponge for miRNA‐7 with its 70 selectively conserved binding sites, resulting in reduced levels of miRNA‐7 expression and increased miRNA‐7‐targeted gene activity.^82^ Additionally, circDOCK1,^83^ mm9_circ_012559,^84^ circ‐SRY^85^ and ZNF609^86^ have similar functions. Furthermore, some circRNAs such as circ‐Mbl,^87^ circ‐PABPN1^88^ and circ‐Foxo3^89^ interact with RNA‐binding proteins other than binding to or releasing miRNAs from their downstream target genes^90^; (2) regulating transcription. For example, circ‐PAIP2 and circ‐EIF3J are reportedly combined with RNA polymerase II complex and translation‐related proteins, resulting in the increased expression of EIF3J and PAIP2^91^, ^92^; (3) translating proteins. Although most circRNAs are regarded as endogenous ncRNAs due to their unique structure,^92^ recent reports have suggested that at least four circRNAs can be translated into proteins. High expression levels of circ‐FBXW7 ^93^ and circ‐SHPRH,^94^ along with proteins programmed by them, are observed in normal human brains, while the expression levels of these proteins are relatively low in glioma. In murine myoblasts, circ‐ZNF609 can also be translated into proteins.^95^ Similar processes are likely to be observed for circ‐Mbl^96^; and (4) regulating epigenetic alterations. On the one hand, several circRNAs have been reported to regulate DNA methylation. CircFECR1, for example, was demonstrated to activate FLI1 by inducing massive CpG DNA demethylation in the promoter of FLI1. In addition, CircFECR1 was reported to decrease the expression of DNMT1 by binding to its promoter and eventually downregulating the level of DNA methylation. Meanwhile, CircFECR1 can also reverse DNA methylation by recruiting TET1 DNA demethylase to the FLI1 promoter in order to induce DNA demethylation.^97^ On the other hand, some circRNAs have been found to regulate methyltransferase EZH2 by interacting with miRNA and subsequently regulating histone methylation indirectly.^98^ For instance, hsa_circ_0020123 and circBCRC4 can promote the activity of EZH2 by sponging miRNA‐144^99^ and miRNA‐101,^100^ respectively.

## 4 THERAPIES TARGETING EPIGENETIC MODIFICATIONS OF CSCS

Given the significant role of epigenetic regulation, it is not surprising that HDAC and DNMT, which play a pivotal role in epigenetic erasers, and writer enzymes have attracted increasing attention and are continuously being studied in the search for cancer therapy.^101^, ^102^, ^103^, ^104^ In addition, the effects of ncRNAs in cancer are also receiving much attention. In this respect, it is natural to think about how we can interfere with the course of events mentioned above, using the corresponding chemical inhibitors.

In order to eradicate all cancer cells, targeting only the primary cancer cells is inadequate. Therefore, it is crucial to target a small population of CSCs. As mentioned above, epigenetic regulation mechanisms are indispensable in the progression of tumour cells and with a better understanding of the epigenome, which provides potential targets for the application of novel therapeutics against different tumour types, it has become increasingly important for us to target these using a variety of specific drugs and inhibitors to improve tumour therapy.

### 4.1 Targeting HDAC and DNMT

Several HDAC and DNMT inhibitors have shown promising effects in different stages of preclinical and clinical trials against diverse cancers. For instance, it is believed that DNMT inhibitors in combination with anti‐TIGIT or anti‐KLRG1 antibodies decrease metastatic potential in keratin‐14^+^ breast cancer cells, which are vulnerable to NK cells.^105^ The eradication of CSCs and the improvement of survival in patients with relapsed and refractory tumours are almost certainly due to the effect of HDAC and DNMT inhibitors.^106^, ^107^ Furthermore, dual inhibitors of DNMT1 and HDAC8 were introduced as novel potential candidates for epigenetic‐based cancer therapeutics for the first time in a recent study.^108^ In addition, trials using the combination of HDAC and DNMT inhibitors for metastatic and/or recurrent non‐small‐cell lung cancer (NSCLC) are currently enrolling patients. The effects of the HDAC inhibitor belinostat are being tested in ongoing trials on recurrent ovarian cancer, along with B‐cell and T‐cell lymphomas.^109^ Furthermore, the effectiveness of HDAC and DNMT inhibitors has not only been shown in trials, but has also shown clinical benefit. DNMT inhibitors in combination with poly (ADP‐ribose) polymerase (PARP) inhibitors have a good effect on recurrent and resistant breast, ovarian and urothelial cancers.^110^, ^111^ Similarly, prolonged progression‐free survival in NSCLC is observed when using a combination of the DNMT inhibitor azacytidine and the HDAC inhibitor benzamidine.^112^ Furthermore, the addition of the HDAC inhibitor panobinostat significantly contributes to better survival of patients with relapsed multiple myeloma who have a treatment regimen combining the proteasome inhibitor bortezomib and dexamethasone.^113^ Given the crucial role of HDAC and DNMT in epigenetic changes, there are accumulating studies focusing on identifying cell types that are sensitive to HDAC and DNMT inhibitors. A genome‐scale network model and gene expression‐based score have been established to predict how metabolic perturbations affect sensitivity to HDAC inhibitors, and the sensitivity of multiple myeloma cells to DNMT inhibitors, respectively.^114^, ^115^

#### 4.1.1 HDAC inhibitors

With the accumulating research on HDAC revealing its mechanisms and functions in tumorigenesis, the potential exploitation and development of HDAC inhibitors for tumour therapy appears promising. The classical pharmacophore of HDAC inhibitors consists of three parts, including a cap structure responsible for interaction with the edge of the entrance of the active pocket within HDACs, a zinc ion‐binding group (ZBG), and a linker that connects the cap and the ZBG, as well as interacting with the hydrophobic tunnel of the active site.^116^ Additionally, the cap can adopt a wide range of structural variations, suggesting that it would be possible to design various HDAC inhibitors with different structures.

The first HDAC inhibitor, vorinostat or SAHA, approved by the US Food and Drug Administration (FDA) in October, 2006 has been used to treat rare cutaneous T‐cell lymphoma. In addition, three other HDAC inhibitors have been approved by the FDA, romidepsin, belinostat and panobinostat, which have primarily been used in multiple myeloma, peripheral T‐cell lymphoma (PTCL) and cutaneous T‐cell lymphoma.^117^ In addition, China has recently approved the benzamide‐based class I HDAC‐selective inhibitor chidamide for the treatment of relapsed or refractory PTCL.^117^

Vorinostat, belinostat and panobinostat are categorized as pan‐HDAC inhibitors that target class I, II, and IV HDACs. In addition, romidepsin inhibits class I HDACs, while class I and IIb HDACs are targeted by chidamide.^118^ Although the expression levels of different HDACs vary among different cancer types and HDAC inhibitors are class‐specific, the effect of inhibitors is still limited. For instance, both romidepsin and vorinostat have been proven to provide efficacy and a lasting response in patients with cutaneous T‐cell lymphoma in Phase 2 multi‐centre trials; however, few of the desired goals were achieved when these two drugs were utilized as single‐agent drugs during the treatment of several solid tumours in clinical trials,^119^, ^120^, ^121^, ^122^, ^123^, ^124^, ^125^, ^126^, ^127^, ^128^, ^129^, ^130^ suggesting that haematological malignancies are more sensitive to HDAC inhibitors, and the combination of HDAC inhibitors and other anticancer drugs and/or radiotherapy may show promise in other cancer treatments.^44^ Importantly, this kind of anticancer drug is associated with several adverse effects, not only those wide‐ranging, easily controlled adverse effects, but also serious and life‐threatening effects such as various cardiac effects, diarrhoea, and myelosuppression. Furthermore, extra caution is needed when such epigenetic modifiers are applied in children whose epigenetic profiles may be associated with their development. Apart from the very modest effect on solid tumours and serious adverse effects, another challenge in the development of novel HDAC inhibitors is that many patients develop resistance to HDAC inhibitors, which is also regularly observed for other types of anticancer drugs.

Recent research shows that HDAC3 is effective for the control of lung alveolar macrophage development and homeostasis, and further evidence indicates that the affected antigen‐presenting function of cluster 3 and the immune‐responsive function of cluster 4 are probably the consequences of loss of HDAC3, suggesting that HDAC3 may play a role in lung cancer.^131^ However, further studies are needed. Thankfully, next‐generation HDAC inhibitors with high selectivity for specific HDAC isoforms are currently being developed ^132^ and include entinostat, a class I HDAC‐selective inhibitor ^133^; NBM‐BMX (NCT03726294), which is specific for HDAC8; and ricolinostat, specific for HDAC6.^134^ These developments expand the potential areas for application of inhibitor treatment by targeting dysregulated HDACs in dormant CSCs.^135^

#### 4.1.2 DNMT inhibitors

As mentioned above, DNMTs are considered promising targets for the epigenetic treatment of tumours; therefore, it is not surprising that DNMT inhibitors have aroused substantial attention in recent years with respect to the regulation of aberrant DNA methylation patterns. Generally, DNMT inhibitors function by inhibiting DNA methylation in order to decrease the level of promoter hypermethylation and enable abnormally silenced tumour suppressor genes such as P15 or CDKN2B, P16 or CDKN2A, MLH1, and RB to re‐express.^136^ There are two types of DNMT inhibitors: nucleoside DNMT inhibitors and non‐nucleoside DNMT inhibitors.^137^ The nucleoside analogues work by incorporating into the DNA and trapping DNMTs to DNA covalently, whereas the non‐nucleoside analogues are capable of targeting the catalytic region of DNMTs to affect their activity.^137^

##### 4.1.2.1 Nucleoside DNMT inhibitors

To date, only two DNMT inhibitors, 5‐azacytidine (azacytidine, Vidaza®, Celgene, Summit, NJ, USA) and 2‐deoxy‐5‐azacytidine (decitabine, Dacogen®, MGI Pharma, Bloomington, MN, USA), both of which are nucleoside DNMT inhibitors, have been approved by the FDA to treat myelodysplastic syndrome (MDS). In 2004, azacytidine, which was administered at a recommended dose of 75 mg/m^2^, administered over a prolonged period of 7 days in a 4‐week cycle, was first proven to be better than best supportive care (BSC) in MDS patients.^138^ According to the data from clinical trials using azacytidine either in combinatorial therapies or as a single agent for a 15‐year period, azacytidine demonstrated less toxicity, but similar or even better overall survival, compared with current AML treatments.^139^ Thus, azacytidine is recommended for AML treatment, especially for elderly patients who cannot bear intensive chemotherapy regimens. Decitabine showed a prolonged median time to progression (TTP) to AML or death, while the overall survival was similar to BSC, and higher cytotoxicity was observed in clinical trials using decitabine either in combinatorial therapies or as a single agent for a 17‐year period.^139^ That being said, the development and approval of these two nucleoside DNMT analogues occurred well before the complexity of methylation patterns had been deciphered.^140^, ^141^ This type of inhibitor is prone to result in a genome‐wide decline of DNA methylation levels and eventually induces the reactivation of genes randomly, including those with potentially deleterious effects. Moreover, nucleoside DNMT inhibitors lack single‐agent efficacy in the treatment of solid tumours, probably because of hypoxia ^142^ and drug infiltration in solid tumours, and are cytotoxic to normal cycling cells. Concerns regarding the specificity and toxicity of nucleoside DNMT inhibitors relate to their intrinsic mechanisms, which hinder their clinical development.^143^ There are currently only two other nucleoside DNMT inhibitors undergoing assessments in clinical trials at present: SGI‐110 in Phase III and 5‐F‐CdR in Phase I.^144^, ^145^ An oligonucleotide antisense inhibitor of DNMT1, called MG98, is also in a Phase I study. Given the shortage of these agents, nucleoside DNMT inhibitors are usually used at low doses to reprogramme and sensitize tumour cells to diverse radiotherapy, chemotherapy and immunotherapy regimens in clinical trials, some of which show good prospects.^112^

##### 4.1.2.2 Non‐nucleoside DNMT inhibitors

Owing to the boundedness of nucleoside DNMT analogues, accumulating research is concentrating on the design and development of non‐nucleoside DNMT analogues. There are five main sources for obtaining novel non‐nucleoside DNMT inhibitors, as follows: (1) repurposing traditional drugs such as procaine,^146^ procainamide,^147^ and hydralazine (currently in a Phase III trial)^148^; (2) natural products such as laccaic acid A,^149^ genistein,^150^ nanaomycin A,^151^ and EGCG (in a Phase II trial at present)^152^; (3) molecules discovered by virtual screening such as DC_05,^153^ NSC137546,^154^ and RG108^155^; (4) molecules identified by experimental high‐throughput screening such as SW155246,^156^ diclone,^157^ and 3‐chloro‐3‐nitroflavanones ^158^; and (5) synthesized molecules such as Δ^2^‐Isoxazoline derivatives,^159^ NSC compound analogues,^160^, ^161^ RG108 analogues^162^, ^163^, ^164^ and SGI‐1027 and its derivatives.^165^, ^166^, ^167^ As for non‐nucleoside DNMT inhibitors other than hydralazine and EGCG, the others are still in the preclinical stages.^144^, ^168^ Although the selectivity and efficacy of novel non‐nucleoside DNMT analogues seem to have improved slightly according to their inhibitory activities (IC_50_/EC_50_), the main weakness in the majority of these agents is the poor DNA demethylation capability in cells and/or relatively low bioactivity at micromolar levels in comparison with the nucleoside DNMT analogues.^153^, ^174^

### 4.2 Targeting ncRNAs

Enormous challenges exist for targeting ncRNAs to develop new drugs for treatment. On the one hand, though the functions and regulatory roles that lncRNAs and circRNAs play in cancer cells make them potential targets for tumour therapeutics, they are still far from being recommended for diagnosis and treatment. As for lncRNAs, there are no feasible or experimental therapies that directly target lncRNAs as yet due to their low expression and the lack of effective tools for adaptation to their particular features.^175^, ^176^ With respect to circRNAs, precise mechanisms related to cancer initiation and progression other than miRNA sponges remain to be elucidated, and more controlled and large‐scale clinical studies are needed before cancer‐specific circRNAs can be applied in clinical practice. That being said, on the other hand, tremendous progress has been made in terms of therapeutics targeting miRNAs, especially in the treatment of various solid tumour types using nanoparticle‐conjugated miRNA mimetics.^177^ In the following section, we focus on miRNA‐based therapeutics.

MiRNAs, if carefully selected, are capable of targeting substantial mRNAs that are altered aberrantly in various cancers. Meanwhile, miRNA‐based therapeutics are feasible owing to advances in technologies to deliver RNA molecules in vivo and provide the chemical modifications to the nucleotide backbone that increase stability and improve targeting to the disease site. MiRNAs can either act as therapeutics in the form of miRNA mimics or as targets of therapeutics in the form of antimiRs.^178^, ^179^, ^180^, ^181^ MiRNA mimics are synthetic double‐stranded small RNA molecules that contain the corresponding miRNA sequence to functionally restore the decreased miRNA expression in cancers. In contrast, the first‐generation antisense oligonucleotide‐based antimiRs have only one strand, designed to target mRNAs or be modified by locked nucleic acids (LNAs). In addition, antimiRs modified with a 2ʹ‐O‐methoxyethyl are also called antagomiRs. These synthetic small RNA molecules have a sequence complementary to miRNAs that affects their function by strongly binding to them.

#### 4.2.1 Replenishing tumour‐suppressive miRNAs

In terms of replenishing tumour‐suppressive miRNAs, miRNA‐34 mimics currently being tested in a phase I clinical trial (NCT01829971) in various haematological and solid tumours are the leading miRNA‐based therapeutics for cancer. In a mouse model of lung cancer, remarkable inhibition of proliferation was observed without adverse effects caused by carrier‐mediated immune stimulation due to the effect of miRNA‐34 mimics encapsulated in lipid nanoparticles.^182^ In addition, lipid nanoparticle‐encapsulated miRNA‐34 mimics also show promising prospects in mouse models of liver^183^ and prostate^184^ cancer. The potential of miRNA‐34 as an anticancer therapeutic has also been demonstrated in other models in several preclinical studies. After miRNA‐34 mimics encapsulated in liposomal carriers had been systemically delivered in an orthotopic model of pancreatic cancer using MiaPaca‑2 cells, tumour growth and CD44^+^ cell counts decreased while tumour cell apoptosis increased, suggesting a decline in metastatic cells.^185^ In addition, in a prostate cancer mouse model, although only a slight reduction in tumour growth was achieved, neutral lipid emulsion‐based strategies to deliver miRNA‐34 mimics still showed promise in the aspect of reduction in metastatic spread to other tissues, as a result of which an increase in survival times was also observed.^184^ In a therapeutically resistant (Kras^LSL‐G12D/+^; Trp53^LSL‐R172H/+^) mouse model of lung cancer, tumour formation and progression were inhibited when treated with miRNA‐34 mimics in vivo.^186^ Furthermore, a combination of miRNA‐34 mimics, let‑7, and the epidermal growth factor (EGFR) inhibitor erlotinib demonstrated synergistic effects in inhibiting the growth of NSCLC cell lines in vitro.^187^

#### 4.2.2 Suppressing oncomiRs

Regarding anticancer strategies based on the suppression of oncomiRs by using antimiRs, several potential target miRNAs express aberrantly. For instance, miRNA‐221 is one of the most remarkably upregulated miRNAs in hepatocellular carcinoma (HCC), leading to the downregulation of key tumour suppressors such as TIMP3, PTEN, and p27^KIP1^.^188^, ^189^ In addition, as a result of the remarkable downregulation of miRNA‐221, increased levels of target mRNA were observed after a cholesterol‐modified form of antimiRNA‐221 was delivered to HCC xenografts intravenously.^190^ In terms of miRNA‐10b, the combination of doxorubicin and miRNA‐10b LNA showed promise in inhibiting metastasis in mouse models of breast cancer.^191^ A recent study has demonstrated that neferine (NEF) significantly suppressed cell proliferation, migration, and invasion, and induced apoptosis by deactivating the PTEN/PI3K/AKT and p38MAPK signal pathways via downregulating miRNA‐10b in the human glioma cell line U251.^192^ MiRNA‐155 is a crucial oncomiR in AML, and a natural product called silvestrol can inhibit colony formation and apoptosis in FLT3‐ITD‐positive (whose overexpression is relevant to poor outcome in AML) AML cell lines, which supports the clinical testing of sivestrol as a novel therapeutic approach for AML.^193^ In addition, miRNA‐630 is overexpressed due to hypoxia in the tumour microenvironment, and exceptional reduction in metastasis and proliferation was observed with the use of antimiR against miRNA‑630 in 1,2‐dioleoyl‐sn‐glycero‐3‐phosphatidylcholine (DOPC) carriers in the orthotopic ovarian tumour model.^194^

#### 4.2.3 Challenges facing miRNA‐based therapeutics and solutions

Although several miRNA‐based therapeutics have shown clinical benefits and a bright future, studies on miRNAs in an effort to achieve a therapeutically effective response in patients are not without their problems. There are five major hurdles in the way of turning miRNAs into drugs: (1) retaining the stability and consistency of miRNAs that are rapidly degraded by RNases in circulation^195^, ^196^; (2) successful delivery of charged nucleic acid analogues across hydrophobic cell membranes to the target tissue^197^; (3) escape from endosomes^197^; (4) preventing off‐target effects and unwanted on‐target effects; and (5) avoiding activation of immune responses.^198^

Fortunately, some of these problems can be effectively solved by modifications. For example, the improved design of double‐stranded miRNA mimic shows less off‐target effects and better efficiency in comparison with single‐stranded miRNA mimics.^199^ In addition, chemical modifications in oligonucleotides such as adding a 2‐OH group,^200^ conjugating a 3‐cholesterol to the passenger strand,^201^ or tagging non‐nucleotide groups to the 3’ end of mimics can markedly increase the stability and activity of the miRNA in serum. Furthermore, in order to increase the amount of antitumour drug uptake by the target site in cancer treatment, the polysaccharide hyaluronic acid can be conjugated to the CD44 marker, which is upregulated in CSCs.^202^, ^203^, ^204^

## 5 CONCLUSION

Despite that the early attempts of cancer therapeutics to target CSCs were disappointing, we have learned much about CSCs and have begun to translate this understanding into the clinic. In this well‐defined clinical context, epigenetic therapies also provide evidence that this strategy against CSCs can be promising and effective. In addition, many potential druggable epigenetic targets remain to be revealed. However, epigenetic modifications are likely to be much more complex than our initial imaginations might suggest. For example, we must consider the existing histone milieu because the cross‐talk between histone modifications has an impact on biological response and protein recruitment when using HDAC inhibitors. In addition, several enzymes thought to function epigenetically may act through non‐epigenetic mechanisms, such as the methylation of cytosolic substrates.

Given this, a clear understanding of the hurdles that inhibit CSC‐targeting epigenetic therapeutics will contribute to the development of better cancer treatments, and these are (1) the properties of many CSCs are not well identified in some cancer types ^205^; (2) the isolated CSCs used in most studies cannot simulate the complex biological microenvironment ^206^, ^207^; and (3) whether CSCs should be activated or dormant in cancer therapy, which currently remains unclear.^208^ Therefore, the future of CSC‐targeting epigenetic therapeutics requires further exploration and substantial effort.

## CONFLICT OF INTEREST

No potential conflicts of interest are disclosed.

## AUTHORS' CONTRIBUTIONS

WBY, YJG and SHZ contributed to conception and design. SHZ contributed to manuscript writing and figures making. YJG revised the figures. CJL, WBY and LJL critically viewed, edited and approved the manuscript. All authors read and approved the final manuscript.

## Data Availability

Data sharing is not applicable to this article as no new data were created or analysed in this study.
